# Foot and Lower Limb Clinical and Structural Changes in Overuse Injured Recreational Runners Using Floating Heel Shoes: Preliminary Results of a Randomised Control Trial

**DOI:** 10.3390/s21237814

**Published:** 2021-11-24

**Authors:** Javier Gamez-Paya, Lirios Dueñas, Anna Arnal-Gómez, Josep Carles Benítez-Martínez

**Affiliations:** 1 Faculty of Health Sciences, Universidad Europea de Valencia, 46010 Valencia, Spain; javier.gamez@universidadeuropea.es; 2 Department of Physiotherapy, Faculty of Physiotherapy, University of Valencia, 46010 Valencia, Spain; anna.arnal@uv.es (A.A.-G.); josep.benitez@uv.es (J.C.B.-M.); 3 Physiotherapy in Motion, Multi-Speciality Research Group (PTinMOTION), Department of Physiotherapy, Faculty of Physiotherapy, University of Valencia, 46010 Valencia, Spain; 4 Exercise Intervention for Health Research Group (EXINH), Department of Physiotherapy, Faculty of Physiotherapy, University of Valencia, 46010 Valencia, Spain

**Keywords:** running, gait retraining, foot, overuse injury, pain management, running footwear

## Abstract

Foot-strike and the associated load rate are factors related to overuse injuries in runners. The purpose of this study was to analyse structural and functional changes in runners using floating heel running shoes, compared with runners using conventional footwear. A randomised control trial was conducted. Twenty runners with overuse injuries were followed over a 12-week gait retraining programme using floating heel running shoes or their conventional footwear. Pain was measured with pressure pain thresholds (PPTs), structural changes were measured with ultrasonography, and severity and impact of injury was scored on the Oslo Sports Trauma Research Centre Overuse Injury Questionnaire (OSTRC-O). Statistical differences were found between groups after the intervention (*p* < 0.001), with a medium size effect SE = 0.8, and the floating heel running shoes group reached higher PPTs values. Participants using floating heel running shoes showed higher OSTRC-O scores than those using their conventional footwear (*p* < 0.05), with higher scores after the intervention (*p* < 0.05). A 12-week gait retraining programme using floating heel running shoes had positive effects on the injury recovery process when compared to the use of conventional footwear, with significant differences in terms of pain and impact on sports activity.

## 1. Introduction

In recent decades, running has become one of the most popular and accessible sports, and the number of runners increases every day [1]. Moreover, running is one of the most efficient ways to achieve physical fitness and has several cardiovascular, muscular, and health benefits [2]. However, running has a relatively high risk of injury, representing a significant problem for runners, since injuries are reported to range between 19% to 79%, [3].

In the lower limbs, the knee and the foot are the most commonly injured locations due to intrinsic factors such as biomechanical abnormalities and muscle functionality [4], or extrinsic factors such as poor running technique [5] or unsuitable running shoes [6]. Overall, the biomechanical effects associated with foot-strike pattern, such as anatomic alignment of lower limb structures [7], stride length or foot pronation, and the greater vertical ground reaction force load rate are thought to be important issues related to several overuse injuries [8,9]. The impact peak is associated with high rates of loading while running [10]. These high vertical force loading rates have been found to be related to a variety of overuse musculoskeletal injuries [11,12], Achilles tendinopathy and plantar fasciitis being two of the most frequently reported [13]. Conversely, the absence of a marked impact peak in the ground reaction force during a forefoot strike compared with a rearfoot strike may contribute to lower rates of injuries in habitual forefoot strikers [5].

The relationship between load rates and running injuries has led to the development of two main strategies to reduce their incidence: gait retraining to lessen running impact [14] and modifying foot strike pattern from rear foot strikes to non-rear foot strikes [15] by using specific footwear in order to change foot-strike pattern [6]. In this sense, previous research has reported that 95% of runners are rear foot strikers (RFS) [16]. The best and safest foot-strike pattern technique is an open discussion topic among experts in running biomechanics. Some studies conclude that there are no differences in the risk of injury between RFS and non-rear foot strikers (NRFS), nevertheless it has been shown that RFS are more likely to experience moderate repetitive stress injury than NRFS [5]. Besides, NRFS pattern has shown to decrease vertical loading rates compared to RFS [17], thus reducing peak vertical loading rates [18]. Therefore, foot-strike pattern is a variable suggested to affect injury rates, and evidence highlights that adopting NRFS running style, specifically midfoot strike pattern, may reduce the likelihood of overuse injury [5]. In this line, a minimalist running shoe with a lug platform can be found on the market. This running shoe aims to change foot strike pattern by promoting non-heel strike landing, therefore reducing energy loss during impact. However, a study analysing their aforementioned effects showed that these shoes may not lead to a landing pattern switch or lower vertical loading rates [19].

A new concept, based on a floating heel model, called the FBR concept (‘Faster and Better Runners’. European Patent EP3061361A4) [20] consists of a running shoe with similar midfoot and forefoot features to conventional designs, but without the midsole under the heel, in order to allow a free vertical movement of the heel without any ground contact, thus taking advantage of the elastic energy provided by plantar flexor muscles. A previous study concluded that FBR promotes a midfoot strike pattern and reduces the impact transient [21]. Another study focused on conventional runners and investigated the kinematic differences between running with conventional shoes or with floating heel running shoes, which allow free movement of the heel without any ground contact during stance [22]. This study concluded that the floating heel shoes encourage running with a non-rearfoot strike pattern and allow lower overstride length and higher heel vertical movement than conventional shoes [22]. In relation to overuse injured runners, Saxelby et al. conducted a case study in which a runner with recalcitrant plantar fasciitis was retrained using floating heel shoes [23], suggesting that this type of footwear could have some potential advantages for injury recovery.

Considering the current gait retraining strategies described above, as well as the biomechanical changes associated with floating heel running shoes (FHS), we hypothesised that including this kind of footwear in a gait retraining process would contribute to the recovery process of the most common overuse running injuries. Therefore, the main objective of this study was to analyse the clinical and structural changes in overuse injured runners using FHS compared to runners using conventional footwear. In particular, the objectives were to study pain pressure thresholds (PPTs) of the injured area, to measure structural changes in tendons and muscles, and to analyse the severity and impact of injury in participants before and after gait retraining.

## 2. Materials and Methods

### 2.1. Participants

**Figure 1:**
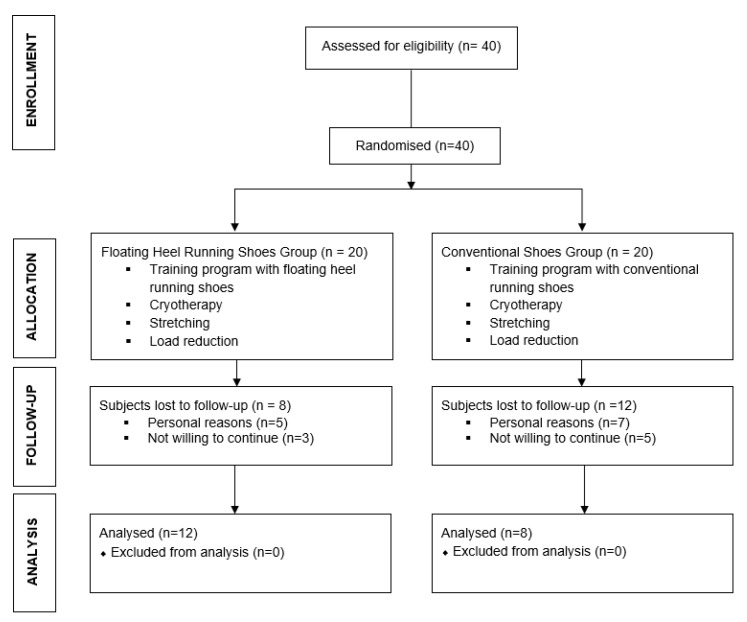
Flow diagram according to CONSORT Statement for the Report of Randomised Trials.

A total of 40 recreational overuse injured runners were recruited from the same running club and screened for possible eligibility criteria. Their mean age was 35.5 ± 8.4 years old (min: 26, max: 59); in relation to gender, 65% were men and 35% women; mean height was 168.6 ± 8.1 cm (min: 156, max: 183); mean weight was 70.8 ± 8.9 kg (min 58, max 93); and BMI was 24.8 ± 1.6 (min 22.9, max 27.7). All of them were included initially in the study, but 20 finished the study and were included in the results (Figure 1). Inclusion criteria were (1) being recreational runners; (2) currently suffering from a lower limb overuse injury; (3) running at least 20 km per week and a personal best time of 5 km in less than 24 min to ensure participants had both experience and commitment to running [21]; and (4) regular RFS (confirmed using high-speed sagittal plane video recording at 100Hz). Exclusion criteria were (1) professional runners; (2) runners using minimalist shoes according to Knapik’s et al. definition [24] (i.e., shoes that provide “minimal interference with the natural movement of the foot, because of their high flexibility, low heel to toe drop, weight and stack height, and the absence of motion control and stability devices.”) or foot orthoses; and (3) previous serious lower limb injuries.

Participants provided informed consent following an explanation of the study’s aims and procedures before entering the study. The present study complies with the Declaration of Helsinki and was approved by the University of Valencia Ethics Committee (H1437042879211).

### 2.2. Study Design

A preliminary randomised controlled clinical trial, with parallel groups and a blinded assessor was carried out from January to April 2019. The outcome measurements were recorded at two assessment times: at baseline, and after the 12-week intervention. First, patients were assessed by an explorer; then, they were randomly allocated into two groups: an experimental group who trained with FHS (FHSG) and a control group who used conventional shoes (CSG). Randomisation was conducted by an external assistant using a random number generator with Statgraphics Centurion XVI software (StatPoint Technologies, Inc., Warrenton, VA, USA). On this basis, the assistant prepared sealed, sequentially numbered envelopes containing the treatment assignments. The post assessment was conducted by the same explorer as in baseline, who was blinded to the baseline examination and to participants’ group assignment. The participants were asked not to make any comments to the explorer during the measurements. Study participants were not blinded to the type of intervention they could receive since they could see and feel the footwear used. Coding, analysis, and interpretation of results was done by another researcher. The study was conducted following the CONSORT extension for pragmatic clinical trials [25].

### 2.3. Intervention

Data were collected at the biomechanical laboratory of the Faculty of Physiotherapy, University of Valencia (Spain). The training sessions took place in the track and field facilities of Valencia city.

**Figure 2:**
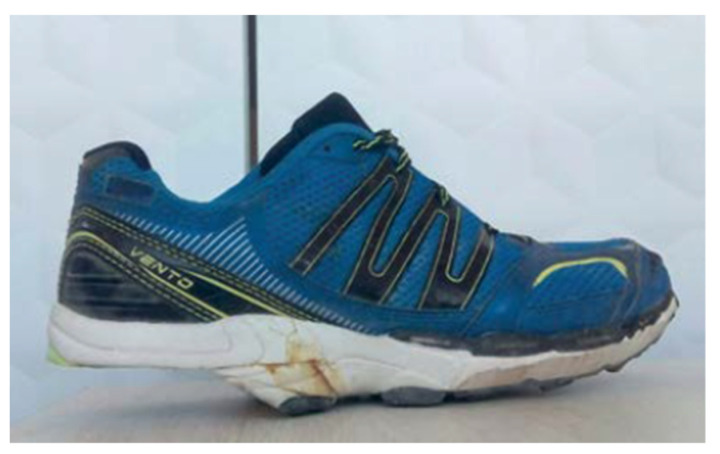
Handmade floating heel shoe.

Participants of both groups followed a 12-week gait retraining programme [14] that consisted of a 2-hour session, 3 days per week. During the first visit, the main researcher manually constructed each runner’s FHS. The heel was removed from a running shoe provided by the participants (Figure 2). FHSG participants then ran for the first time with the FHS. First, the research team took footage of each runner on a treadmill (Supplementary Material: Videos S1 and S2). Second, the videos were analysed together with the participants, and the main researcher gave them verbal feedback in order to achieve a proper running technique which, following Souza et al.’s criterion [26], is defined by midfoot strike pattern, vertical tibia in the moment of contact, trunk lightly leaned forward, and short overstride.

In the first part of the session, runners started with 30 min of warm up (15 min of slow running and 15 min of running drills). The participants then began the running training. A 45% load (mileage) reduction was set in the first week (considering the load that every runner followed prior to injury). Then, the load was increased 5% each week, until reaching their usual individual training load at week 12. Each group wore different types of shoes:

- FHSG: followed a training programme to get used to FHS. Through the 12-week intervention the amount of FHS running was increased progressively. From week 1 until week 8, the runners combined both types of running shoes: conventional footwear and FHS. From week 9 until the end of the study, the runners used FHS only. Table 1 shows the training program followed by the FHSG runners.
- CSG: trained in the same way, but using their conventional footwear.

After running, both groups received cryotherapy (20 min/session) and performed stretching exercises (20 min/session). During the programme, a running coach (J.G.P.) with more than 20 years of experience conducted a follow-up of each runner in order to check that they were properly adapting to the new shoe and running technique. The treatments were applied independently in relation to time frames and settings. The runners were asked not to practice any other sport, nor to follow any additional treatment.

### 2.4. Outcomes

All outcome measurements were recorded at baseline (pre) and after the 12-week intervention (post). One blinded physiotherapist (J.C.B.M.) with 20 years of clinical experience performed all the pre- and post-intervention assessment measurements.

#### 2.4.1. Pressure Pain Thresholds (PPTs)

**Figure 3:**
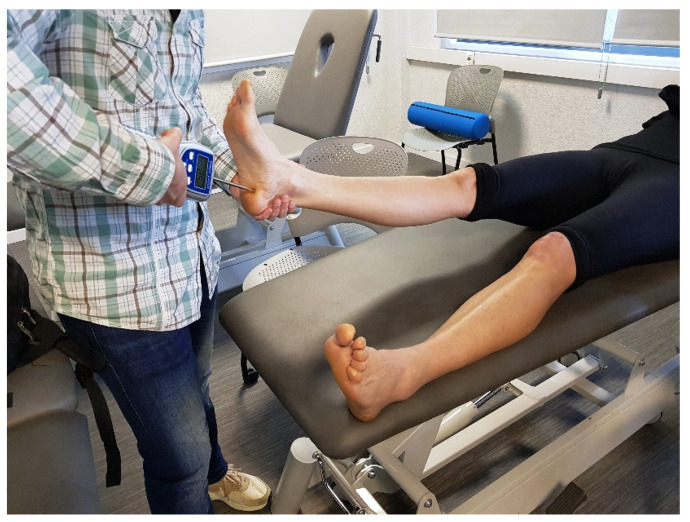
Pressure pain thresholds measurement at the plantaris fascia’s tender point.

The minimal pressure (kg/cm^2^) that induces pain was measured using an algometer (Pain Test-Model FPK 40; Wagner Instruments, Greenwich, CT, USA). The measurement of the PPTs was performed with participants in a standardised position depending on the injured area and following a previous published protocol [27]. The device was positioned at the point where the runner referred to feeling the greatest tenderness, using a rubber disc with a surface area of 1 cm^2^. The algometer was pressed against the selected area, and the pressure was increased by 1kg/s until the patient experienced the first painful sensation [28] (Figure 3).At this point, the peak force of the measurement was displayed on the algometer. Three measurements were applied on the same spot, with 30 s rest between them. The mean of the three measurements was taken for analysis [29]. The reliability of the pressure algometry procedure has been found to be high (ICC = 0.91 (95% confidence intervals (CI): 0.82, 0.97)) for healthy subjects [30]. Pressure algometry measures have also been shown to be reliable and valid in patients with a variety of musculoskeletal pain syndromes [31].

#### 2.4.2. Ultrasonography

All participants were instructed to lie down on a bench with their legs relaxed in a supine or prone position. Muscles of the dominant leg and/or affected structure were analysed. The parameters of the image capture system were standardised and kept constant during all measurements for the same muscle or structure and in all participants, in order to compare changes between sessions preventing the influence of bias on such differences. All measurements were conducted twice and the mean value calculated.

For all participants, the muscle thickness was measured in the rectus femoris (RF), vastus intermedius (VI), vastus lateralis (VL), and hamstrings (H) (Supplementary Material: Table S1 [32,33,34,35,36]), using a brightness mode (B-mode) ultrasonographic apparatus (8300 CHISON, Wuxi, China), with a linear probe 38 mm in width and an excitation frequency of 5–10 MHz. Ultrasound settings such as frequency, focal depth, image depth, power, and gain were optimised to best identify the collagenous tissue that defines the outer border of the muscle and the depth of the muscle. Great care and a generous amount of gel was used to ensure that minimal pressure was applied to the muscle tissue when an image was scanned.

**Figure 4:**
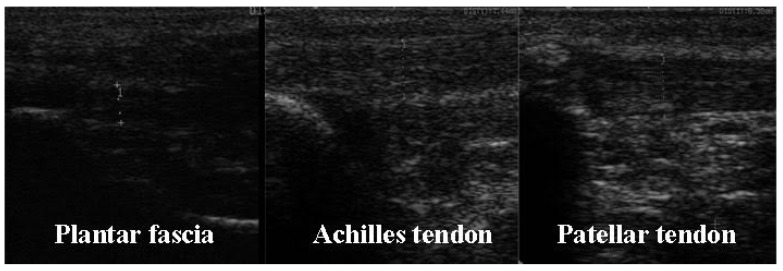
Images of the ultrasound scanning in the Plantar fascia, Achilles tendon, and Patellar tendon.

When the injured area was located in the patellar tendon (PT), Achilles tendon (AchT), or plantaris fascia (PF), the ultrasound scanner was applied in the affected structure. The ultrasound device settings were optimized to best identify the border of the tendon or fascia and gel was used to avoid ultrasound refraction on the skin. Figure 4 shows an example of the ultrasonography images obtained.

#### 2.4.3. Oslo Sports Trauma Research Centre Overuse Injury Questionnaire (OSTRC-O)

This questionnaire was developed by Clarsen et al. [37] to record overuse injuries and their severity and impact. It consists of four questions: question 1 and 4 have four response options, and questions 2 and 3 have five response options. The four questions record the level of sports participation (question 1), training volume (question 2), sports performance (question 3), and pain (question 4) during the past 7 days. Questions 1 and 4 scored 0-8-17-25, and questions 2 and 3 scored 0-6-13-19-25; the response value, 0 represented no problems and 25 meant the maximum level of difficulties and problems running. The term “problems” refers to pain, ache, stiffness, swelling, instability/giving way, locking, or other complaints related to the injured body segment [37].A severity score for each overuse injury was then calculated from 0 to 100 based on these four questions, with 0 representing no impact and 100 representing the maximum severity and impact on sports activity [37]. Each participant answered the OSTRC-O twice, during the first (pre) and second (post) visits. The Spanish version of the OSTRC-O has shown good reliability and validity in youth athletes (i.e., aged between 12 and 18 years old) and excellent internal consistency (Cronbach’s alpha = 0.88 (95% CI, 0.86–0.90), ICC = 0.85 (95% CI, 0.81–0.89)) [38].

#### 2.4.4. Statistical Analysis

Firstly, a Shapiro–Wilk test was conducted to analyse the normality of the data (*p* < 0.05). Secondly, descriptive statistics were calculated for each variable depending on its normality: mean and standard deviation for parametric variables, and median and interquartile range for non-parametric variables.

When parametric assumptions were not met, we compared pre- and post-conditions using Wilcoxon rank-sum test. To study the differences between groups after the intervention (post-condition), we used the Mann–Whitney U test. A 0.05 level of significance was adopted. Lastly, we determined the size effect using the approach proposed by Rosenthal for non-parametric variables [39] (low effect: 0.1; medium effect: 0.3; large effect: 0.5).

$$r=\frac{z}{\sqrt{N}}$$

Conversely, if the data met the parametric assumptions, an independent *t*-test between-group statistical comparison was conducted. Effect sizes were determined for all parametric variables to aid in the interpretation of any trends found. The effect size was calculated as the difference between the two group means divided by the pooled standard deviation and was defined as small (d = 0.2), medium (d = 0.4), and large (d = 0.8) [40]. The statistical analysis was performed using SPSS Statistics for Windows (IBM SPSS Statistics for Windows, Version 26.0. IBM Corp, Armonk, NY, USA).

## 3. Results

Of the 40 prospective participants, 20 dropped out due to reasons nonrelated with the study, and 20 participants completed the 12-week programme (FHSG n = 12; CSG, n = 8) (Figure 1). They trained a mean of 3.8 ± 1.2 days/week, with a training experience of 11.4 ± 4.6 years. All runners belonged to the same running club, so they were following the same training programme. The type of overuse injuries for each group are presented in Table 2.

**Figure 5:**
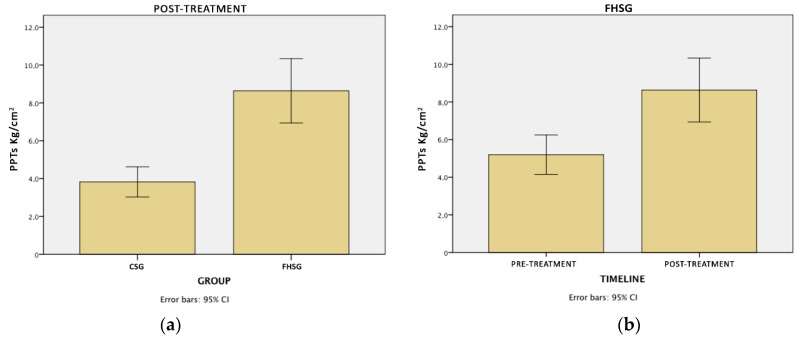
PPT measurements with significant differences. (**a**) Mean differences between groups at the end of the intervention (post-treatment). (**b**) Mean differences within the floating heel running shoes group (FHSG) between the baseline and the end of the intervention (pre- and post-treatment).

PPT measurement showed no differences at baseline between groups, whereas we found statistical differences between groups after the intervention (*p* < 0.001), with a medium size effect SE = 0.8, and with the experimental group reaching higher PPTs (Table 3). There were also differences in the within-group comparison of the FHSG between pre- and post-conditions, showing that after the intervention, the FHSG bore more pressure on the overuse injured area (*p* < 0.001; SE = 1.1) (Figure 5).

Ultrasonography scans showed structural changes in some muscles and tendons. The FHSG showed a reduction of the cross-sectional area of the quadriceps muscles (RF, VI and VL) after treatment, as can be seen in Table 3. There were significant changes in the within-group comparison of the CSG for the AchT cross-sectional area, with higher values at the end of the gait retraining programme (pre: 5.0 ± 1.4 mm; post: 6.0 ± 1.4 mm; *p* < 0.05; d = 0.8 (Table 3).

Regarding the analysis of OSTRC-O, the Wilcoxon rank-sum test showed differences between pre-and post-conditions in the FHSG group. The runners showed higher values before (Mdn = 73.5 ± 24.5) than after the intervention (Mdn = 43 ± 13), z = −3.05, *p* < 0.05, r = 0.68, whereas no differences were found in the CSG between pre- (Mdn= 76.6 ± 4.5) and post- (Mdn = 67.5 ± 14.5) conditions, z = −1.86, *p* > 0.05. After the training program, we found differences between groups; the FHSG showed a lower score (Mdn = 47 ± 13) than the control group (Mdn = 67.5 ± 14.5), z = −3.4, *p* < 0.05, r = 0.76 (Table 4).

## 4. Discussion

The results of this preliminary study showed that recreational runners with overuse injuries can benefit from the floating heel concept when training. A 12-week gait retraining programme together with the use of floating heel running shoes had positive effects on the injury recovery process when compared to the use of conventional footwear, with significant differences in pain thresholds and impact on sports activity.

In the present study, significant changes in pressure pain thresholds (PPTs) were found after the programme, with the experimental group (FHSG) reaching higher PPTs than the control group (CSG), with a mean difference between groups of 4.81. Moreover, the increase observed in the FHSG was over the minimum clinically important difference (i.e., greater than 1.77 kg/cm^2^ [30]). These findings may suggest that the modification of the landing technique related to the floating heel shoe towards a non-rear foot striker could be of relevance in order to reduce pain related to overuse injuries of the lower limbs. Thus, overuse injuries may be related to loading activities and this mechanical overuse may lead initially to inflammation and, with time, to degeneration [41]. By modifying the force load, this inflammation and the nociceptive changes produced may have been reduced. This aspect highlights the idea that by using floating running shoes, the vertical force loading rates may be reduced, thus decreasing the impact peak and allowing the healing of the injured tissue.

Structural changes were also found in the ultrasonography study of muscle and tendon structures. The changes observed in muscle thickness were variable. While measured bellies of the quadriceps in the FHSG revealed a significant decrease in the transverse section of rectus femoris, vastus intermedius, and vastus lateralis bellies (with mean differences of −1.9, −2.3, and −2.0, respectively), this did not occur in the hamstrings. These results may imply that the quadriceps muscles work less with floating heel footwear. Indeed, the literature reports that with a midfoot-strike pattern, the force moment in the knee is lower [42], as well as the overstride [22] compared to that produced in a rear foot-strike pattern associated with conventional shoe running form. The fact that a muscle slightly decreases in diameter could be understood as an adaptation of the body to the loads to which it is being subjected. Accordingly, gait retraining effects achieved with floating heels, such as midfoot-strike pattern and shorter overstride, could lead to a lower stress on the quadriceps, and thus, on the knee joint. Studies with larger samples are warranted to obtain conclusive results.

Otherwise, tendon thickness showed a similar behaviour. While its thickness did not change in the patellar tendon, the Achilles tendon underwent a thickness increase in the CSG (mean difference of 1.08). An increase in thickness has been observed in pathological tendons [43,44], and Achilles tendon compression happening at end range dorsiflexion has been associated with insertional Achilles tendinopathy [45]. The fact that the rear foot strike technique performed by the CSG is related to high dorsiflexion and that the non-rear foot strike technique performed by the FHSG is related to low dorsiflexion during stance [17] could explain the differences between groups in subjects in whom this structure is affected. 50% of the CSG patients suffered from Achilles tendinopathy. Within this group, a trend of decreased PPTs and increased Achilles tendon thickness was observed after the 12-week intervention. These aspects could highlight why recreational rear foot-strike runners with an Achilles tendon injury take time to recover if they continue to run without gait retraining [14] and using their conventional footwear [46]. Further research with a larger sample size is warranted.

In relation to OSTRC-O values, we would like to highlight that our results showed a high effect size (r = 0.8). The minimally important change of the OSTRC-O severity score, that is, the smallest change score which is truly important to the runner, is estimated to be 18.5 for injured (half) marathon runners [47]. In the current study, the participants from the FHSG showed OSTRC-O scores exceeding the minimally important change, both in the between-group comparison (20.5) and the within-group comparison (26.5). This suggests that the training had an outstanding effect on severity and impact of injury.

Over the last years, researchers have attempted to determine the clinical effectiveness of eccentric training for tendon injuries, with promising results for chronic Achilles tendinosis [48,49], patellar tendinopathy [49], and plantar fasciitis [50], among others. Our results are consistent with other studies that also used eccentric contractions from an injury rehabilitation perspective. The study by Alfredson et al. [51], with a similar sample as that of our study (15 recreational running athletes with Achilles tendon pain and a mean age of 44 years), applied an eccentric-resistance exercise programme with progressively increasing loads. After 12 weeks, all subjects returned to preinjury levels of running activity, whereas subjects in the conventional resistance exercise group ultimately required surgery. Similar findings have also been reported when using eccentric training as part of a resistance exercise programme in recreational athletes (aged 15–50 years) with knee tendinosis [52,53]. In our study, patients from the FHSG were able to avoid transient force impact through the eccentric loading of the posterior calf muscles, significantly reducing this load [11]. These eccentric exercises may have achieved a clinically and statistically meaningful pain reduction for our participants.

Forces that are repeatedly applied to the body could lead to positive remodelling of a structure if the forces fall below the tensile limit of the structure and if sufficient time elapses between force applications [12]. High-load strength training that causes high loads across tendons are promoted as being beneficial for degenerative tendon disorders, with eccentric exercises receiving the most attention [54]. Increasing evidence supports that eccentric training, which has been underused and undervalued, may also help to prevent musculoskeletal injury and overcome musculoskeletal impairments [55]. The question remains how to incorporate this type of training in a safe way in an athlete’s programme. The key to avoiding any adverse response, such as damage to the muscle, lies in the dose [55]. If the magnitude and duration of the force is increased gradually over time, no symptoms of damage, swelling, or even soreness are present [56]. Programme design is important for any athlete’s performance and success. We believe one of the strengths of the present study, and possibly the reason for its positive results, was the individualised progression of both the overall load and mileage and the specific training load wearing floating heel shoes.

Our study included both physical therapists and a running coach, which enhances the idea of adopting a multidisciplinary approach to the treatment and management of sports injuries. By including not only these professionals, but also general practitioners, podiatrists, and even sport psychologists, the re-training process could be improved and adherence to rehabilitation programmes could be further increased [57].

To our knowledge, this is the first study analysing the effects of floating heel shoes in injured recreational runners. Despite several decades of running shoe development aimed at reducing injuries, the incidence of overuse injuries has remained relatively unchanged [58]. In recent years, clinicians’, podiatrists’, and researchers’ efforts have been focused on designing running footwear adaptations aimed at reducing the incidence of overload injuries in runners [6], improving their sport performance [59,60], and analysing foot movement and posture [61]. To date, a 12-week gait retraining programme using floating heel running footwear has obtained promising results.

### 4.1. Limitations

This study has several limitations that need to be acknowledged. First of all, the main limitation of this study was the small sample size and its heterogeneity regarding the injured area. Given the small sample size, definitive conclusions cannot be extracted; hence further replication in a larger sample is warranted. The relatively small sample size may also be a potential reason for the lack of significant differences found for some outcomes. A larger sample size could allow the adequate balancing of groups regarding gender, age, BMI, and injury. Statistical power analysis (G*Power 3.1.9) estimated that a sample of 25 participants per group was needed to achieve a 0.8 study power [62]. Another limitation of this study is the high dropout rate during the intervention period. A high dropout rate was expected, since the intervention had a period of 12 weeks. Moreover, no rewards were given to participants, there was a lack of positive effects in the control group, and Easter holidays were during the study period. The authors acknowledged that this could have resulted in bias, and this should be taken into account for future studies, by, for instance increasing the sample size and trying to avoid holiday periods. Furthermore, we have not performed a kinetic and kinematic analysis, but have only relied on kinetic variables. Future studies are needed in which a full biomechanical analysis is performed. Additionally, reliability and validity studies were not conducted in the ultrasonographic muscle and tendon assessment, although it was carried out by an assessor with more than 15 years of experience in the use of ultrasonography and widely documented references have been taken from the literature.

### 4.2. Suggestions for Further Research

Based on our findings we suggest broadening the spectrum of lower limb overuse injuries including, for instance, iliotibial band syndrome, tibial stress fractures, and patellofemoral pain syndrome. Larger sample sizes studies are warranted for this purpose.

Furthermore, analysing the long-term adaptation of skeletal muscle morphology and architecture to the floating heel running technique using new technologies such as 3D ultrasound imaging technology could be a future line of research.

## 5. Conclusions

The use of a floating heel running shoe together with a 12-week gait retraining programme has positive effects on the injury recovery process in a group of recreational runners with various typical overuse running injuries, by decreasing both pain and impact on their sports activity. Modifying the footwear towards a non-rear foot striker, together with continuous eccentric stimuli during running, could be of relevance for lower limb recovery in cases of overuse injury. Further research with a larger sample size for verification of the study results is warranted.

## Figures and Tables

**Table 1:** Adaptation training with floating heel running shoes.

Week	Number of Sessions	Training
1	3	3 sets of 3 min with FHRS, 2 min rest
2	3	3 sets of 5 min with FHRS, 2 min rest
3	3	3 sets of 6 min with FHRS, 2 min rest
4	3	3 sets of 8 min with FHRS, 2 min rest
5	3	2 sets of 12 min with FHRS, 2 min rest
6	3	2 sets of 15 min with FHRS, 2 min rest
7	3	2 sets of 20 min with FHRS, 2 min rest
8	3	2 sets of 25 min with FHRS, 2 min rest
9	3	100% of the session running with FHRS
10	3	100% of the session running with FHRS
11	3	100% of the session running with FHRS
12	3	100% of the session running with FHRS

FHRS: Floating heel running shoes.

**Table 2:** Type of overuse injuries for each group.

Type of Injury	FHSG (n = 12)	CSG (n = 8)
Plantar fasciitis	5 (42)	1 (13)
Achilles tendinopathy	3 (25)	4 (50)
Patellar tendinopathy	2 (17)	3 (38)
Iliotibial band syndrome	2 (17)	0 (0)

Data are shown as frequency and percentage (%). FHSG: floating heel shoe group; CSG: conventional shoe group.

**Table 3:** Pressure pain thresholds and ultrasonography results.

Measures		Time Mean ± SD		Mean Differences and Size Effect (*d*)	
		Pre	Post	Within-Group Differences	Between-Group Differences (Post)
PPTs (kg/cm^2^)	FHSG	5.2 ± 2.4	8.6 ± 3.8	3.4 ^†^; d = 1.1	4.8 ^†^; d = 0.8
	CSG	4.7 ± 1.5	3.8 ± 0.8	−0.8	
Ultrasonography (mm)					
Rectus femoris	FHSG	22.5 ± 3.3	20.6 ± 4.3	−1.9 *; d = 0.5	1.3
	CSG	20.4 ± 3.7	19.3 ± 3.3	−1.1	
Vastus intermedius	FHSG	21.8 ± 3.9	19.5 ± 4.1	−2.3 *; d = 0.6	3.1
	CSG	17.0 ± 4.2	16.4 ± 5.0	0.6	
Vastus lateralis	FHSG	24.7 ± 4.9	22.7 ± 4.7	−2.0 *; d = 0.4	2.2
	CSG	21.7 ± 4.7	20.6 ± 4.1	−1.1	
Hamstrings	FHSG	29.8 ± 4.6	30.9 ± 3.4	1.2	−0.1
	CSG	31.3 ± 4.0	31.0 ± 3.7	−0.3	
Patellar tendon	FHSG	4.5 ± 0.7	4.2 ± 0.7	−0.3	0.4
	CSG	4.0 ± 0.5	3.8 ± 0.5	−0.2	
Achilles tendon	FHSG	5.3 ± 1.5	5.3 ± 1.7	−0.0	0.8
	CSG	4.9 ± 1.4	6.0 ± 1.4	1.1 *; d = 0.8	
Plantaris fascia	FHSG	3.2 ± 1.3	2.6 ± 0.6	−0.5	−0.1
	CSG	2.5±0.4	2.8±0.8	0.3	

FHSG: Floating heel shoe group; CSG: conventional shoe group; SD: Standard deviation; PPTs: pressure pain thresholds; *p* value was calculated with *T* test: * *p* < 0.05; ^†^ *p* < 0.001; Cohen’s d effect size was calculated for comparisons where statistically significant differences were obtained.

**Table 4:** OSTRC-O results.

Measures		Time Median (Interquartile Range)		*p* Value Wilcoxon Test and Size Effect (*r*)	*p* Value U Mann-Whitney and Size Effect (*r*)
		Pre	Post	Within-Group Differences	Between-Group Differences (Post)
OSTRC-O score	FHSG	73.5 (24.5)	47 (13)	*p* < 0.01 r = 0.7	*p* < 0.001 r = 0.8
	CSG	76.6 (4.5)	67.5 (14.5)	*p* > 0.05	

Effect size was calculated for comparisons where statistically significant differences were obtained. Low effect: 0.1; medium effect: 0.3; large effect: 0.5 [39].

## Data Availability

All available data can be obtained by contacting the corresponding author.

## Supplementary Materials

The following are available online at https://www.mdpi.com/article/10.3390/s21237814/s1, Video S1: Man running with floating heel running shoes. Midfoot strike pattern. Video S2: Man running wearing conventional shoes. Rear foot strike pattern. Table S1. Details of the ultrasound scanning.
