# Supplementary material for: Foot and Lower Limb Clinical and Structural Changes in Overuse Injured Recreational Runners Using Floating Heel Shoes: Preliminary Results of a Randomised Control Trial
Source: Sensors (Basel). 2021 Nov 24;21(23):7814. doi: 10.3390/s21237814 (PMC8659959; doi:10.3390/s21237814)
Supplement: Supplementary file 1 [file sensors-21-07814-s001.zip › Supplementary Table S1.pdf]

sensors

MDPI

Article

Foot and Lower Limb Clinical and Structural Changes in Overuse Injured Recreational Runners Using Floating Heel Shoes: Preliminary Results of a Randomised Control Trial

Javier Gamez-Paya [1,†], Lirios Dueñas [2,3,*,†], Anna Arnal-Gómez [2,3] and Josep Carles Benítez-Martínez [2,4]

[1] Faculty of Health Sciences, Universidad Europea de Valencia, 46010 Valencia, Spain; javier.gamez@universidadeuropea.es
[2] Department of Physiotherapy, Faculty of Physiotherapy, University of Valencia, 46010 Valencia, Spain; anna.arnal@uv.es (A.A.-G.); josep.benitez@uv.es (J.C.B.-M.)
[3] Physiotherapy in Motion, Multi-Speciality Research Group (PTinMOTION), Department of Physiotherapy, Faculty of Physiotherapy, University of Valencia, 46010 Valencia, Spain
[4] Exercise Intervention for Health Research Group (EXINH), Department of Physiotherapy, Faculty of Physiotherapy, University of Valencia, 46010 Valencia, Spain
* Correspondence: lirios.duenas@uv.es; Tel.: +34-963-983-853 (ext. 51264)
† These two authors contributed equally to this work.

Supplementary Table S1. Details of the ultrasound scanning.

Muscle	Measurement Parameters and References
Quadriceps	Using the superior pole of the patella as the distal reference point and the anterior superior iliac spine as proximal, the thickness of RF, VI and VL was measured at 50% of this distance [32]. These locations were based on data from Kawakami et al. [33], and represent the location of the maximum cross-sectional of each individual quadriceps muscle. To account for the lateral location of VL, measurements were taken 10% of thigh circumference in the medial direction from the midpoint of the thigh. The vertical diameter of each muscle was measured on the inner edge of the muscle.
Hamstrings	To locate the measurement sites of the hamstring muscles, an anthropometric tape measure was placed along the length of the thigh from the greater trochanter to the lateral condyle of the femur. One point was marked with a marker pen at 60% from the greater trochanter and the probe was placed on the medial posterior aspects of the thigh, which represented the maximal cross-sectional area of this muscle [34]
Patellar tendon	The subject was in supine position with approximately 30° knee flexion, obtained by placing a small pillow beneath the popliteal space. The tendons visualized in the longitudinal view and scans were recorded at the widest thickened point in the sagittal plane [35].
Achilles Tendon and plantaris fascia	the tendons were measured on the middle of the medial malleolus determined by palpation. This site was chosen because it was an easy way to find bony landmark that could be used for all participant's tendons. The AchT was measured with the subjects in the prone position and the foot pointing straight down. The PF was measured with participant in supine position and the foot pointing straight up. The measurements were performed in a short-axis scan with the transducer perpendicular to the fibre direction. After maximal enlargement, the thickness was determined using on-screen callipers and the cross-sectional area using the trackball [36]. The thickness was found as the longest anterior-posterior diameter.

32. Giles, L.S.; Webster, K.E.; McClelland, J.A.; Cook, J. Can Ultrasound Measurements of Muscle Thickness Be Used to Measure the Size of Individual Quadriceps Muscles in People with Patellofemoral Pain? *Phys. Ther. Sport Off. J. Assoc. Chart. Physiother. Sports Med.* **2015**, *16*, 45–52, doi:10.1016/j.ptsp.2014.04.002.
33. Kawakami, Y.; Muraoka, Y.; Kubo, K.; Suzuki, Y.; Fukunaga, T. Changes in Muscle Size and Architecture Following 20 Days of Bed Rest. *J. Gravitational Physiol. J. Int. Soc. Gravitational Physiol.* **2000**, *7*, 53–59.
34. Abe, T.; Loenneke, J.P.; Thiebaud, R.S. Ultrasound Assessment of Hamstring Muscle Size Using Posterior Thigh Muscle Thickness. *Clin. Physiol. Funct. Imaging* **2016**, *36*, 206–210, doi:10.1111/cpf.12214.
35. Benítez-Martínez, J.C.; Valera-Garrido, F.; Martínez-Ramírez, P.; Ríos-Díaz, J.; Del Baño-Aledo, M.E.; Medina-Mirapeix, F. Lower Limb Dominance, Morphology, and Sonographic Abnormalities of the Patellar Tendon in Elite Basketball Players: A Cross-Sectional Study. *J. Athl. Train.* **2019**, *54*, 1280–1286, doi:10.4085/1062-6050-285-17.
36. Brushøj, C.; Henriksen, B.M.; Albrecht-Beste, E.; Hölmich, P.; Larsen, K.; Bachmann Nielsen, M. Reproducibility of Ultrasound and Magnetic Resonance Imaging Measurements of Tendon Size. *Acta Radiol. Stockh. Swed. 1987* **2006**, *47*, 954–959, doi:10.1080/02841850600854936.
